# DNA and histones impair the mechanical stability and lytic susceptibility of fibrin formed by staphylocoagulase

**DOI:** 10.3389/fimmu.2023.1233128

**Published:** 2023-08-17

**Authors:** Erzsébet Komorowicz, Veronika J. Farkas, László Szabó, Sophie Cherrington, Craig Thelwell, Krasimir Kolev

**Affiliations:** ^1^ Institute of Biochemistry and Molecular Biology, Department of Biochemistry, Semmelweis University, Budapest, Hungary; ^2^ Plasma Chemistry Research Group, Institute of Materials and Environmental Chemistry, Research Centre for Natural Sciences, Budapest, Hungary; ^3^ South Mimms Laboratories, Medicines and Healthcare Products Regulatory Agency, Potters Bar, United Kingdom

**Keywords:** staphylocoagulase, fibrin, fibrinolysis, NET, histone, extracellular DNA

## Abstract

**Background:**

Staphylocoagulase (SCG) is a virulence factor of *Staphylococcus aureus*, one of the most lethal pathogens of our times. The complex of SCG with prothrombin (SCG/ProT) can clot fibrinogen, and SCG/ProT-induced fibrin and plasma clots have been described to show decreased mechanical and lytic resistance, which may contribute to septic emboli from infected cardiac vegetations. At infection sites, neutrophils can release DNA and histones, as parts of neutrophil extracellular traps (NETs), which in turn favor thrombosis, inhibit fibrinolysis and strengthen clot structure.

**Objectives:**

To characterize the combined effects of major NET-components (DNA, histone H1 and H3) on SCG/ProT-induced clot structure, mechanical and lytic stability.

**Methods:**

Recombinant SCG was used to clot purified fibrinogen and plasma. The kinetics of formation and lysis of fibrin and plasma clots containing H1 or core histones+/-DNA were followed by turbidimetry. Fibrin structure and mechanical stability were characterized with scanning electron microscopy, pressure-driven permeation, and oscillation rheometry.

**Results:**

Histones and DNA favored the formation of thicker fibrin fibers and a more heterogeneous clot structure including high porosity with H1 histone, whereas low porosity with core histones and DNA. As opposed to previous observations with thrombin-induced clots, SCG/ProT-induced fibrin was not mechanically stabilized by histones. Similarly to thrombin-induced clots, the DNA-histone complexes prolonged fibrinolysis with tissue-type plasminogen activator (up to 2-fold). The anti-fibrinolytic effect of the DNA and DNA-H3 complex was observed in plasma clots too. Heparin (low molecular weight) accelerated the lysis of SCG/ProT-clots from plasma, even if DNA and histones were also present.

**Conclusions:**

In the interplay of NETs and fibrin formed by SCG, DNA and histones promote structural heterogeneity in the clots, and fail to stabilize them against mechanical stress. The DNA-histone complexes render the SCG-fibrin more resistant to lysis and thereby less prone to embolization.

## 1 Introduction

*Staphylococcus aureus* is currently the most lethal infectious agent in the developed world, attempts to develop a protective vaccine have failed so far (1). Although some *Staphylococci* are harmless co-habitants in our body, pathogenic species, such as *S. aureus* can cause not only non-severe skin and soft tissue infections, but abscesses, and life-threatening pneumonia, sepsis, or endocarditis, as well. *S. aureus* has been recently emerging as the most frequent bacterial cause of infective endocarditis (2, 3). Whilst an improvement in oral health and dental prophylaxis may have contributed to the decline in *Streptococcal* diseases, an observed increase in contracting *S. aureus* may be attributed to an increasing number of patients with permanent catheters (used for dialysis or cancer treatment) and with prosthetic heart valves. This shift in the underlying pathogen led to an overall 18% in-hospital mortality of infective endocarditis, with 28% mortality in the *S. aureus* group vs 10-18% in other groups of bacterial infective endocarditis. Different mortality among different bacterial background may be in part due to variations in the affected patient populations, but poor outcome was associated with neurological complications, such as higher frequency of ischemic stroke due to emboli from *S. aureus*-infected cardiac vegetation, as well as of brain hemorrhage, which was more frequent in patients on anticoagulants (4). Almost 20% of patients with infective endocarditis have developed sepsis and septic shock, associated with a 4-fold increase in mortality (5).

On the host side, innate immune response seems to play a major role in the combat against the bacteria, whereas pathogenic *Staphylococci* express several virulence factors to successfully manipulate the host’s hemostatic system (1, 6). Activated neutrophils release neutrophil extracellular traps (NETs) containing sticky DNA strands decorated with histones and antimicrobial proteins, which can entangle bacteria and favor fibrin formation and platelet activation (7–10). The resulting coagulum is to seal off pathogens and enhance their killing, before they can spread out. On the side of *S. aureus*, however, there are common virulence factors, such as staphylocoagulase (SCG) and von Willebrand factor-binding protein (VWbp), which can form a 1:1 stoichiometric complex with human prothrombin and insert their N-termini into the activation pocket of prothrombin leading to an active protease termed “Staphylothrombin” (11). Staphylothrombin converts fibrinogen to fibrin, which together with platelets, is incorporated as a crucial component in endocarditis vegetations, and abscesses (1, 12). Several experimental data suggest that the bacteria can hide and multiply behind this fibrin barrier: turning off any virulence factor related to this barrier formation increases survival rate in experimental animal models (1, 6).

We have previously described the structural characteristics, as well as the mechanical and lytic stability of fibrin and plasma coagulum generated by Staphylocoagulase/Prothrombin (SCG/ProT) in order to address the molecular background of the high rate of septic emboli from cardiac vegetations (13). SCG/ProT formed a more porous, less shear stress-resistant fibrin network, which was more susceptible to tissue-type plasminogen activator (tPA)-induced lysis, all of which could favor clot disintegration. In our present study we investigated how the two major NET-components, histones and DNA, which have been found to strengthen the fibrin network and prolong fibrinolysis (10, 14–16), affect the stability of the SCG/ProT-induced clots.

## 2 Materials and methods

Human fibrinogen (plasminogen free), histones from calf thymus of type IIIS containing predominantly H1 histone (H1) and type VIIIS containing predominantly arginine-rich core histones, as well as calf thymus DNA were purchased from Merck KGaA (Darmstadt, Germany). The chromogenic substrates for plasmin Spectrozyme-PL (H-D-norleucyl-hexahydrotyrosyl-lysine-p-nitroanilide) were from BioMedica Diagnostics (Windsor, NS Canada). Bovine thrombin purchased from Serva (Heidelberg, Germany) was further purified by ion-exchange chromatography on sulfopropyl-Sephadex yielding preparation with specific activity of 2,100 IU/mg (17) and 1 IU/ml was considered equivalent to approximately 10.7 nM by active site titration (18). Recombinant SCG was prepared, characterized, and used at a 1:1.2 molar ratio with prothrombin (ProT; from human plasma, Merck KGaA) to form the SCG/ProT complex, as described previously (13). Plasminogen and plasmin were prepared as previously described (14). Recombinant tPA was from Boehringer Ingelheim, Ingelheim am Rhein, Germany. Heparins used were unfractionated heparin (UFH, Heparin Sodium, Wockhardt, Wrexham, UK) and low molecular weight heparin (LMWH, Enoxaparin, NIBSC reagent code 11/174, 275 anti-IIa IU and 1030 anti-FXa IU per ampoule, 9.4 mg/ampoule, NIBSC, S Mimms, UK). The heparin pentasaccharide fondaparinux sodium (HPS) was the product of GlaxoSmithKline, London, UK.

### 2.1 Characterization of the structure of fibrin clots

*Scanning electron microscopy (SEM)* was used for the measurement of fiber thickness in fibrin. Fibrin clots were prepared in duplicates: 7.4 μM fibrinogen in 10mM HEPES buffer pH7.4 containing 150 mM NaCl (HBS) and 75 mg/l histones or 50 mg/l DNA alone or combined was clotted with thrombin or SCG/ProT at 2nM for 2 h at 37°C (the clotting process has reached a plateau by 2 hours, as verified with turbidimetry measurements). Clots were processed for SEM imaging as detailed previously (19) and images were taken with scanning electron microscope EVO40 (Carl Zeiss GmbH, Oberkochen, Germany). SEM images were analyzed to determine the distribution of fibrin fiber diameters using self-designed program functions running under the Image Processing Toolbox v.11.7 of MatlabR2023a (The Mathworks, Natick, MA, USA) as previously described (19–21). The diameter of 300 fibers was measured on 4-6 SEM images. Clots were characterized by the median and low-bottom quartile of the diameter values.

*Fluid permeability assays* were adapted from previously described methods to characterize clot porosity (22, 23). Fibrin clotting was initiated by mixing 5 nM thrombin or SCG/ProT and 8.8 μM fibrinogen in HBS with or without 75 mg/l histones and 50 mg/l DNA and 25 µl clotting mixture was transferred to a channel in an uncoated IBIDI µ-slide VI 0.4 (Ibidi GmbH, Gräfelfing, Germany) and the slides were incubated in a humidity chamber at 37°C for 2 h to allow for complete clotting. Thereafter, 10 µl HBS containing 0.01% Tween-20 (HBS-T) was added to the clot-loading orifice and 100 µl HBS-T was added to the opposite end to exclude bubble formation while attaching a 2 ml Luer syringe for the buffer reservoir. The syringe was filled up to the top with HBS-T resulting in a 5 cm high fluid column, whereas clots containing H1 histone alone were perfused with a 2.5 cm high fluid pressure head due to repeatedly observed structural collapse at the 5 cm pressure head. Following a 30 min wash of the clots with the permeation buffer, liquid was removed from the opposite, outlet end of the channel, and then, every 30 min for 120 min (or every 15 min for 60 min in the case of highly permeable clots) the volume of throughput liquid was calculated from its weight (using 1 g = 1 ml). The fluid pressure head was kept constant by refilling the buffer reservoir with HBS-T. Pore size of the fibrin clots was estimated on the basis of the permeation co-efficient (Darcy’s constant, Ks): $K_{s}=\frac{Q\times n\times L}{t\times A\times \Delta P}$ , where, Q = volume of permeated buffer (cm^3^); n= viscosity of buffer (10^-2^ poise = 10^-7^ N.s/cm^2^); L= length of clot (1.2 cm); t= time (s); A= cross-sectional area of clot (1.52x10^-2^ cm^2^); ΔP= Pressure drop (0.049 N/cm^2^ or its half for fibrin/H1 clots). Each experiment was carried out with 3 parallels in one IBIDI µ-slide, handling 4 sets at a time, repeated at least 3 times on different days.

*Viscoelastic properties of the fibrin clots* were studied with oscillation rheometry, as previously described (19). Fibrinogen (7.4 μM in HBS) was pre-mixed with 25 µg/ml histones or 100 µg/ml DNA or their combination and immediately after the addition of 8 nM thrombin or SCG/ProT the clotting mixture was transferred to the stationary plate of HAAKE RheoStress1 oscillation rheometer (Thermo Scientific, Karlsruhe, Germany). The cone (Titanium, 2° angle, 35 mm diameter) of the rheometer was brought to the gap position and an oscillatory shear strain (γ) of 0.015 at 1 Hz was imposed at 2 min after clot initiation. Measurements of storage modulus (G’) and loss modulus (G’’) were taken for 15 min with HAAKE RheoWin data manager software v.3.50.0012 (Thermo Scientific). Following this 15-min clotting phase, the flow limit of the fibrin gels was measured in the same samples increasing the applied shear stress (τ) from 0.01 to 1000 Pa stepwise in 300 s and the resulting strain was used for calculation of the viscosity modulus (η). The gel-fluid transition in the fibrin structure was indicated by an apparent fall in viscosity, which point could be characterized by two parameters: the maximal bearable strain (γ_max_) preceding the abrupt fall in viscosity and the critical shear stress *τ_0_* that resulted in γ_max_.

### 2.2 Turbidimetric assays

Fibrin formation and dissolution were followed by measuring the light absorbance at 340 nm at 37°C with a CLARIOstar spectrophotometer (BMG Labtech, Ortenberg, Germany).

*Fibrinolysis with tPA incorporated in fibrin.* Fibrinogen (6 μM) and 15 nM plasminogen in HBS containing 75 µg/ml histones or 50 µg/ml DNA or their combination, was mixed with 8 nM thrombin or SCG/ProT and 1.3 nM tPA in the wells of 96-well microtiter plates. In these experimental setups clotting and tPA-mediated fibrinolysis are co-occurring processes, hence, care was taken to avoid incomplete clotting due to premature fibrinogen degradation. Maximal turbidity values were within a +/-10% range of A_max_ values reached without the lytic agents. Both H1 and core histones, as well as the DNA were tested at various concentrations in the 0-200 µg/ml range, and they all caused a dose-dependent increase of A_max_ values without reaching a saturation. Hence, histones and DNA were applied at concentrations resulting in submaximal, but measurable effects. Based on previous observations on circulating plasma levels of histones and DNA in human patients, these chosen concentrations are also relevant local concentrations at the sites of SCG/ProT-action (24, 25).

*Extrinsic fibrinolysis with surface-applied tPA.* Clots were pre-formed in the microplate wells by clotting fibrinogen (6 μM) containing 150 nM plasminogen in the absence or presence of the above listed additives with 5 nM thrombin or SCG/ProT. After 2 hours of clotting (by which time clot turbidity reached a plateau), 15 nM tPA in HBS-T was layered on the clot surface to initiate plasminogen activation and fibrinolysis.

*Formation and extrinsic lysis of plasma clots*. Citrated normal plasma was supplemented with 1 μM plasminogen, recalcified with equal volume of 25 mM CaCl_2_ and clotted with 9 nM thrombin or SCG/ProT in the presence of the following additives, alone or in various combinations: 0.25 g/l histones, 0.1 g/l DNA, heparins at the *in vivo* relevant therapeutic concentrations (2.5 mg/l UFH, 2.5 mg/l LMWH, or 0.5 mg/l HPS). Turbidity of the developing clot was followed in the microplate reader, and at 60 min, when turbidity curves have all reached a plateau, lysis was initiated by layering 100 nM tPA on the clot surface.

For quantitation of the turbidimetric fibrinolytic experiments, clotting time (CT_90_ for fibrin or CT_50_ for plasma clots) was defined as the time needed to reach 90% or 50% of the maximal turbidity, A_max_, on the ascending part of turbidimetric curves. Lysis time was defined as the time elapsed from time zero (intrinsic lysis) or from the time of tPA addition (extrinsic lysis) until the turbidity of the clot was reduced to the half (LT_50_) of its maximal value, on the descending part of turbidimetric curves.

### 2.3 Statistical analysis

The distribution of the data on fiber diameter was analyzed according to an algorithm used previously (19, 21): theoretical distributions were fitted to the empirical data sets and compared using Kuiper’s test and Monte Carlo simulation procedures. The statistical evaluation of other experimental measurements in this report was performed with Kolmogorov-Smirnov test (Statistics and Machine Learning Toolbox v.12.5 of MatlabR2023a).

## 3 Results

### 3.1 The impact of NET-components is stronger on the structure of SCG/ProT- clots than on thrombin-induced clots

**Figure 1:**
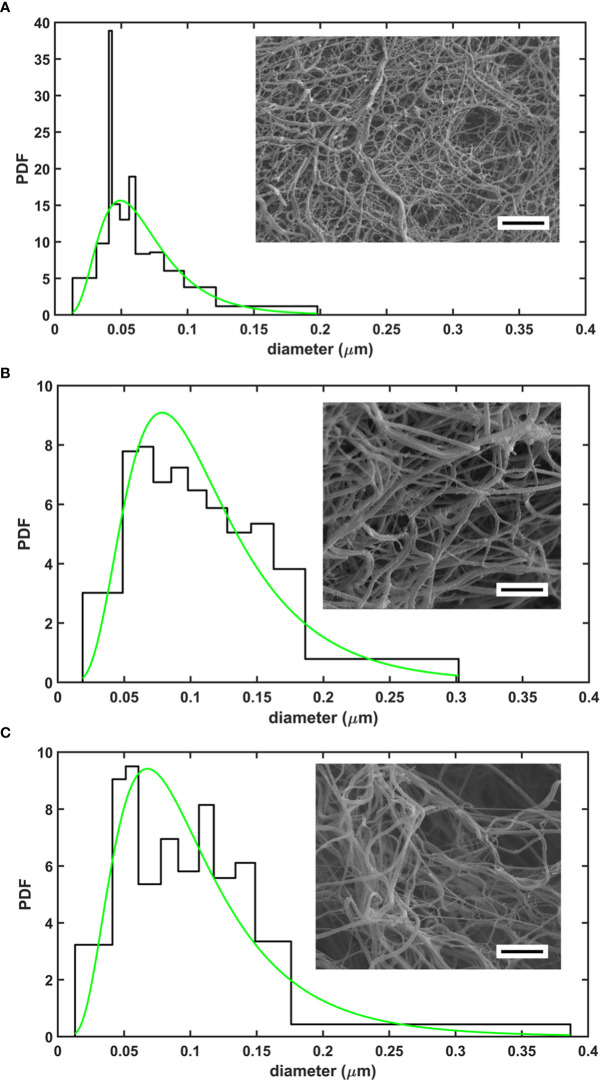
Structure of SCG/ProT-induced clots evaluated with scanning electron microscopy. Fibrin fiber distribution patterns in various clot types with a representative image are shown for fibrin formed in the absence **(A)**, or in the presence of 75 µg/ml H1 histone **(B)**, or 75 µg/ml H1 histone combined with 50 µg/ml DNA **(C)**. Note the more heterogeneous structure with thicker fibers enclosing larger pores in the presence of H1 histone+/-DNA. Quantitative analysis of 4-6 images/clot type was performed as detailed in *Materials and methods* (scale bar is 2 µm). The probability density function (PDF) is shown for the empiric (histogram) and the fitted lognormal (green line) distributions of the diameter values. The numeric parameters of the fitted distributions are summarized in **Table 1**.

A general trend to increase the fibrin fiber diameter and pore size in SCG/ProT-induced clots was observed for all examined NET-components except for isolated DNA (**Table 1**). The thickest fibers and the highest porosity were found with H1 histone (66% increase in diameter, and a 10-fold increase in Ks), and DNA at the applied concentration partially reversed the H1-effects. Fibers were 25% thicker in clots with core histones, and DNA generally favored the formation of a tighter network, albeit its effect was not always statistically significant. The presence of NETs in fibrin led to a broader distribution pattern of the fibrin fiber diameter, which indicates a more heterogeneous clot structure (**Figure 1**). Compared to network parameters of clot-analogues induced by thrombin (**Table S1** in the online **Supplemental Material**), SCG/ProT-induced fibrin networks were typically less porous, except in the presence of H1 histones, which was 2-fold more porous than most of the thrombin-induced NET/fibrin clots.

**Table 1:** Structural characteristics of SCG/ProT-generated fibrin in the presence of NET components.

	none	DNA	H1 histone	H1 histone + DNA	core histone	core histone + DNA
fiber diameter	61 (45–84)	58 (42-80)	100* (72-140)	91*# (63-131)	81* (58-114)	82* (60-112)
K_s_ Darcy constant	1.75 (0.20)	1.33 (0.20)	19.91* (2.23)	4.66*# (0.42)	1.29 (0.18)	0.90* (0.11)
maximal turbidity	1.00 (0.02)	1.16* (0.02)	6.49* (0.17)	3.16*# (0.42)	1.75* (0.08)	1.50*# (0.06)

Fibrin clots containing histone H1 or core histone (75 µg/ml) alone or in combination with 50 µg/ml DNA were examined for fibrin fiber diameter (scanning electron microscopy, median (top, bottom quartile)), network porosity (fluid permeability, K_s_ Darcy constant 10^-9^ cm^2^, mean (SEM)), and fiber mass/length ratio (maximal turbidity, A_max_, mean (SD)) in relative units compared to pure fibrin). Asterisks and pound signs by K_s_ and A_max_ values indicate *p*<0.05 according to Kolmogorov-Smirnov test in the comparison of fibrin containing NET-components to pure fibrin, and of histone-DNA to fibrin with histone alone, respectively, n=12-20. Asterisks and pound signs by fiber diameter values indicate different distributions in the Kuiper’s test.

### 3.2 NET-components tend to destabilize SCG/ProT-induced composite clots

**Figure 2:**
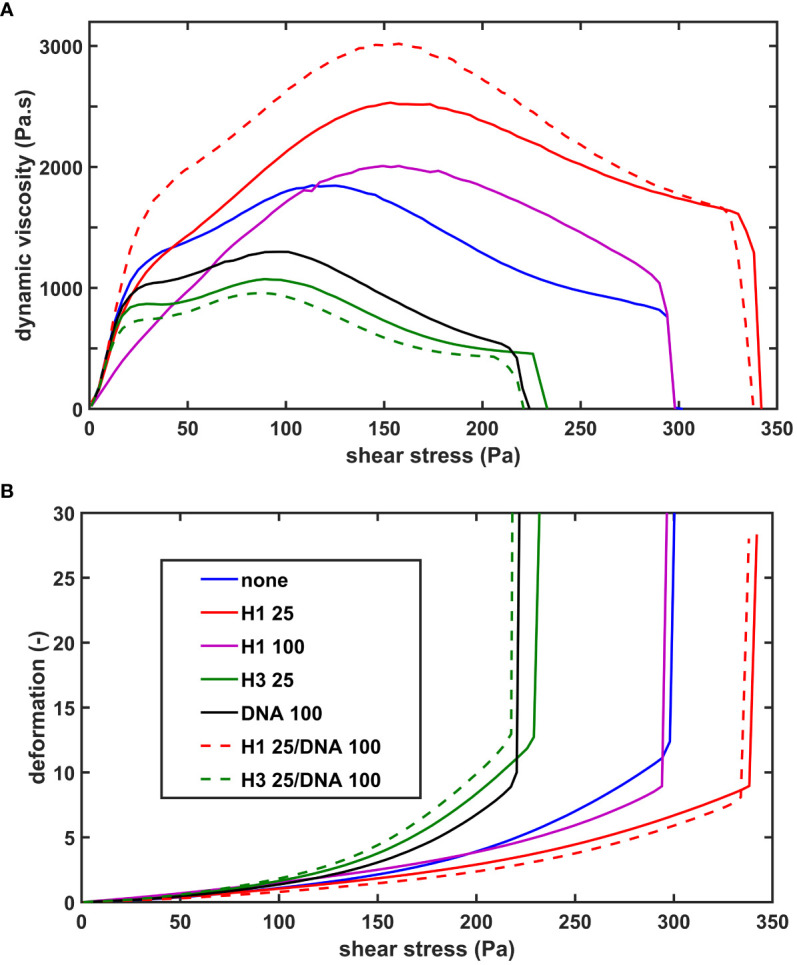
The effect of NET-components on the shear resistance of SCG/ProT-induced fibrin. Fibrinogen without (none) or with supplemented NET- components (H1 histone, core histones (H3), or DNA alone or combined at the indicated concentrations in µg/ml, (see inset box) was clotted with SCG/ProT under superimposed periodic oscillatory deformation in the measurement gap of the oscillation rheometer, as detailed in *Materials and Methods*. After the 15 min clotting phase an increasing shear stress *τ* was applied to the same clot and the resulting relative deformation γ was measured. Representative flow curves of the deformation γ **(B)** and the corresponding computed dynamic viscosity (*η*) **(A)** are presented for the various clot types, whereas numerical data with statistics are summarized in **Table 2**.

We evaluated the effects of histones and DNA on the viscoelastic behavior of composite fibrin clots using oscillation rheometry (**Figure 2**; **Table 2**.). The NET components were applied at concentrations used in several previously published studies (10, 16). DNA at 100 µg/ml mildly softened the fibrin structure, as suggested by the decreased critical shear stress (τ_0_) at which gel/fluid transition occurred. Core histones alone, or in combination with DNA did not significantly alter the mechanical properties of the clot. The previously seen robust H1 histone-mediated strengthening of thrombin-induced clots (26) was less pronounced in terms of critical shear stress (τ_0_) of the SCG/ProT-fibrin and accompanied by different changes of the storage and loss moduli. H1 histones at 25 µg/ml did not significantly increase the storage and loss moduli. As we further increased the H1 concentration to 100 µg/ml, the fibrin network turned significantly softer and less elastic, as indicated by the decreased storage and loss moduli (G’ and G”), and increased loss tangent, and these effects were counteracted by the applied DNA.

**Table 2:** Viscoelastic parameters of SCG/ProT-induced fibrin clots containing histones and DNA.

	none	DNA	H1 histone	H1 histone 100 µg/ml	H1 histone + DNA	core histone	core histone + DNA
G’ storage modulus (Pa)	105.9 (4.6)	99.5 (7.2)	106.5 (2.4)	79.5* (3.6)	101.6 (7.1)	105.2 (5.5)	101.3 (9.6)
G’’ loss modulus (Pa)	7.8 (0.6)	6.7* (0.7)	7.7 (0.4)	6.5* (0.5)	7.3 (0.4)	6.5 (0.5)	6.9* (0.3)
G’’/G’ loss tangent (-)	0.073 (0.004)	0.068 (0.005)	0.072 (0.003)	0.082* (0.004)	0.074 (0.007)	0.062* (0.003)	0.068 (0.006)
τ_0_ critical shear stress (Pa)	309.9 (19.8)	283.7* (6.4)	355.5* (20.5)	297.8 (3.7)	311.3# (18.0)	315.2 (29.4)	288.1 (27.8)
ϒmax, maximal deformation (-)	11.2 (1.6)	9.0 (1.6)	8.8* (1.4)	9.2 (0.4)	9.7 (1.5)	10.2 (1.8)	9.9 (0.6)

Fibrin clots containing H1 or core histone (25 µg/ml, unless otherwise indicated), or DNA (100 µg/ml) alone or in various combinations were examined by oscillation rheometry, as detailed in Materials and methods. The plateau values of the storage modulus (*G’*), loss modulus (*G’’*) and loss tangent (G’’/G’) at the end of the 15 min clotting phase, as well as the critical shear stress (τ_0_) at the maximal bearable relative deformation (γ_max_) before the gel/fluid transition in the fibrin structure are presented as mean (standard deviation). Asterisks and pound signs indicate *p*<0.05 according to Kolmogorov-Smirnov test in comparison to pure fibrin, or to fibrin containing only histone of the same type, respectively, n=4-8.

### 3.3 Kinetics of formation and lysis of SCG/ProT-induced composite fibrin clots containing histones and DNA

**Figure 3:**
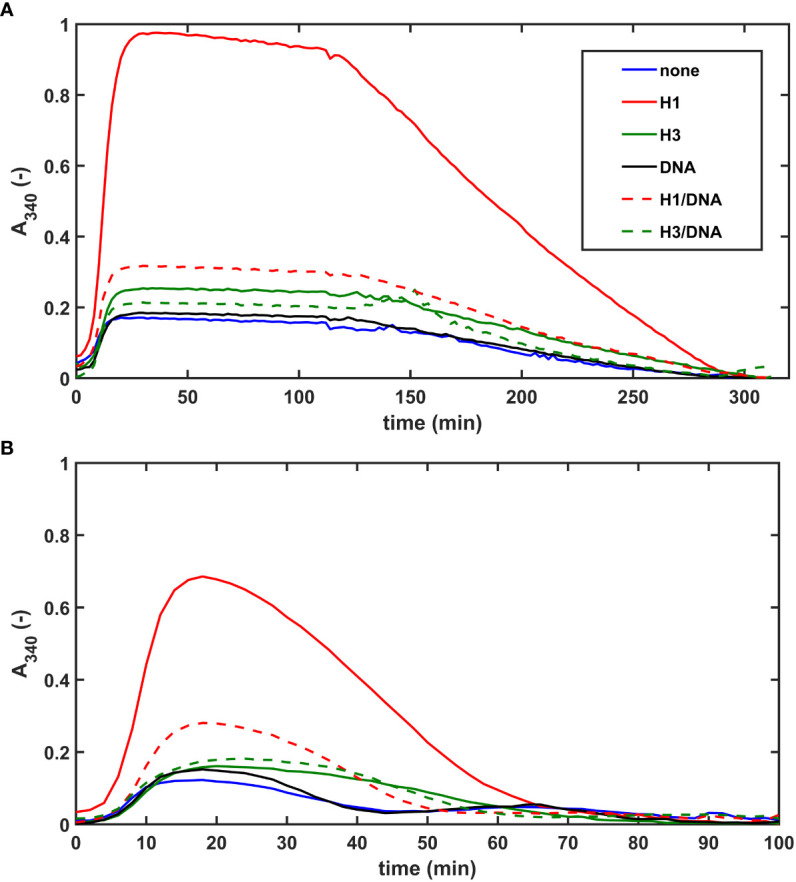
NET-components confer fibrinolytic resistance to SCG/ProT-clots in both intrinsic and extrinsic tPA-mediated models. **(A)** Extrinsic lysis model: fibrinogen containing 150 nM plasminogen and 75 µg/ml H1 or core histones (H3), or 50 µg/ml DNA alone, or combined as indicated was clotted in microplate wells with 8 nM SCG/ProT for 120 min and thereafter tPA (15 nM) was applied to the surface of the clot to trigger lysis. **(B)** Intrinsic lysis model: fibrinogen containing 15 nM plasminogen and histones and/or DNA as in panel A was clotted with 8 nM SCG/ProT in the presence of 1.3 nM tPA to induce fibrinolysis simultaneously with clotting. Ascending and descending parts of the turbidimetric curve recorded as absorbance at 340 nm wavelength indicate fibrin formation and dissolution. Presented kinetic curves are averaged from 4 parallel measurements on the same day, and numerical data shown in **Table 3**; **Table S2** in the online **Supplemental Material** were calculated from at least 3 experiments performed on different days.

In our extrinsic lysis model, the dissolution of preformed fibrin clots containing NET-components and plasminogen was initiated with tPA applied on the surface of the clot. The maximal turbidity (A_max_) of SCG/ProT-induced pure fibrin was 6-fold lower than that of thrombin-induced fibrin, and this difference was diminished in the presence of H1 (**Figure 3A**; **Table 3**). Both H1 and core histones, as well as DNA increased the A_max_ for both SCG/ProT and thrombin-induced clots, and histone-effects were counteracted by DNA at the applied concentration (**Table 3**). Although A_max_ values suggested subtle differences between thrombin and SCG/ProT-induced network structures, fibrinolysis modulation by incorporated NET-components was very similar: core histones prolonged lysis-times by 40%, whereas combinations of DNA with histones by 20-30%.

**Table 3:** NET-components confer fibrinolytic resistance to both SCS/ProT-, and thrombin-mediated clots.

		none	DNA	H1 histone	H1 histone + DNA	core histone	core histone + DNA
Thrombin	Amax	0.925 (0.058)	1.054* (0.014)	1.267* (0.027)	0.403*# (0.044)	1.248* (0.057)	1.082*# (0.016)
	LT50	1.00 (0.02)	1.07 (0.02)	1.10 (0.03)	1.24* **(0.06)**	1.40* **(0.05)**	1.23*# **(0.0.02)**
SCG/ProT	Amax	0.152 (0.023)	0.176* (0.003)	0.986* (0.026)	0.480*# (0.064)	0.266* (0.012)	0.228*# (0.009)
	LT50	1.00 (0.02)	1.07 (0.02)	1.04 (0.02)	1.29*# **(0.07)**	1.39* **(0.05)**	1.19*# **(0.03)**

Fibrin clots containing 150 nM plasminogen and the NET-components histone H1 or core histone (75 µg/ml) alone or in combination with 50 µg/ml DNA were pre-formed with 5 nM SCG/ProT or thrombin. Lysis was initiated with tPA (15 nM) applied to the clot surface after 120 min clotting, when all clot-types reached a plateau of maximal turbidity, Amax (mean (SEM)). Lysis-time (LT50) was defined as the time needed to reach half-maximal turbidity at the descending end of the curve, measured from the time of tPA-addition. LT50 values (mean (SEM)) are presented in relative units compared to pure fibrin. Asterisks and pound signs indicate *p*<0.05 according to Kolmogorov-Smirnov test in the comparison of fibrin containing NET-components to pure fibrin, and of histone-DNA to fibrin with histone alone, respectively, n=12-20. Markedly prolonged lysis time values associated with the presence of NET components are shown in bold.

The lower maximal turbidity for SCG/ProT-induced fibrin and its dramatic correction by H1 were observed in our intrinsic fibrinolysis model, too (**Figure 3B**). When both tPA and plasminogen were incorporated into the fibrin clot, DNA slightly prolonged the lysis-time, which was further prolonged by H1 in combination with DNA. Core histones caused a 2-fold or 1.5-fold prolongation in lysis-time of thrombin-, or SCG/ProT-induced clots, respectively, and the prolonged lysis-times were not significantly dampened by DNA.

### 3.4 Effects of histones and DNA on the formation and lysis of SCG/ProT-induced plasma clots and their modulation by heparin derivatives

**Figure 4:**
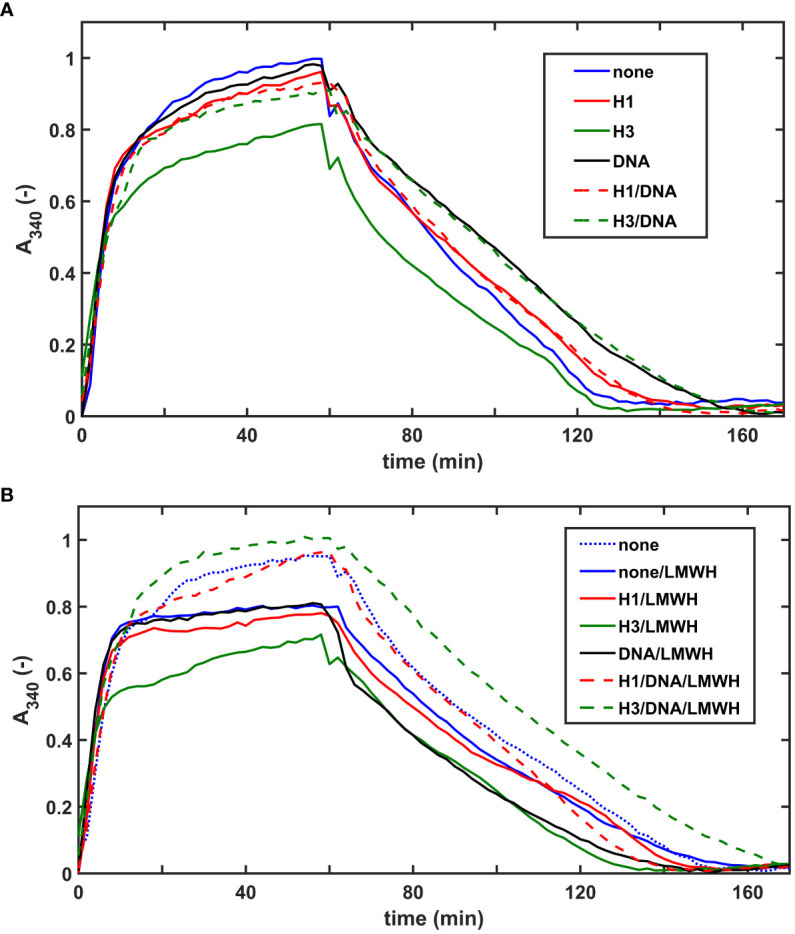
Effects of NET-components and heparins on the dissolution of pre-formed SCG/ProT-plasma clots. SCG/ProT-mediated plasma clots containing 1 µM plasminogen and 250 µg/ml H1 or core histone, or 100 µg/ml DNA, alone or combined were pre-formed in microplate wells in the absence **(A)** or presence **(B)** of 2.5 µg/ml LMWH (curve for pure plasma clot is also presented in panel B for comparison). Clot development was followed by turbidimetry as absorbance at 340 nm, and fibrinolysis was initiated at 60 min by applying 100 nM tPA on the clot surfaces. Presented kinetic curves are averaged from 4 parallel measurements on the same day, and numerical data shown in **Table 4** were calculated from at least 3 such experiments performed on different days.

We evaluated the effects of NET-components also in a plasma milieu, where we included the heparin-type anticoagulants UFH, LMWH, HPS at concentrations relevant for the clinical practice. As opposed to thrombin-induced plasma clotting, SCG/ProT could catalyze plasma clotting even in the presence of UFH, since staphylothrombin is not sensitive to inhibition by antithrombin. Clotting time values (CT_50_ in **Table S3**, online **Supplemental Data**) indicated that heparins could not delay plasma clotting in the absence or presence of NET-components, moreover 30-40% shortening was observed in certain cases, such as UFH or HPS alone, or combining one histone-type with UFH or HPS. Heparins favored plasma clotting when added with DNA alone or with DNA combined with core histones. Fibrin fiber properties, reflected indirectly in the maximal clot turbidity (A_max_ in **Table S3**, online **Supplemental Data**) mirror the speed of clot formation: the lowest A_max_ values are mostly concordant with acceleration of clotting (shorter CT_50_), but when combined with DNA, heparins accelerate clotting without a significant A_max_ change. DNA, alone or combined with core histones prolonged the tPA-mediated lysis of plasma clots by 20-30%, whereas core histones alone rendered the clots easier to dissolve (**Figure 4A**; **Table 4**). Both UFH and LMWH could counteract the DNA-mediated inhibition of clot lysis, but none of the heparin derivatives could do so in clots containing core histones and DNA together (**Table 4**). Taken the frequent administration of LMWH in critically ill patients, it might be of clinical interest that SCG/ProT-induced plasma clots containing LMWH alone, or in combination with NET-components, were typically associated with 10-20% shorter LT_50_ values than their non-LMWH counterparts, and the fastest lysis-times (20-30% shorter than controls) were found in the presence of core histones, alone or combined with heparins (**Figure 4B**; **Table 4**).

**Table 4:** tPA-mediated lysis-time of pre-formed plasma clots containing NET-components and heparins.

	none	H1 histone	core histone	DNA	H1 histone + DNA	core histone + DNA
no heparin	1.00 (0.03)	0.98 (0.03)	0.84* (0.04)	1.34* **(0.04)**	0.93 (0.03)	1.20* **(0.04)**
UFH	0.80* (0.01)	1.05 (0.05)	0.75* (0.06)	0.65*# *(0.07)*	0.99 (0.04)	1.22* **(0.04)**
LMWH	0.86* (0.06)	0.81*# *(0.08)*	0.68*# *(0.05)*	*0.79#* *(0.05)*	*1.09#* *(0.06)*	1.28* **(0.05)**
HPS	1.06 (0.2)	0.70*# *(0.15)*	0.76* (0.01)	1.24* **(0.11)**	0.84* (0.08)	1.18* **(0.08)**

Plasma clots containing 1 µM plasminogen and 250 µg/ml H1 or core histone, or 100 µg/ml DNA, alone or combined were pre-formed in microplate wells in the absence or presence of heparins (2.5 µg/ml UFH or LMWH, or 0.5 µg/ml HPS). Development of clot structure was followed by turbidimetry at 340 nm wavelength, and clot lysis was initiated with 100 nM tPA applied to the clot surface at 60 min, when all curves have reached their plateaus. Lysis-time was defined as the time needed to reach half-maximal turbidity measured from the time of tPA-addition. Values (mean (SEM)) are presented in relative units compared to clots without any additive. Asterisks indicate *p*<0.05 according to Kolmogorov-Smirnov test when comparing clots containing NET-components+/-heparins to pure plasma clot, whereas pound signs indicate a *p*<0.05 significant heparin-effect in comparison with the heparin-free respective plasma/NET composite clot. Markedly prolonged values associated with the presence of NET components are shown in bold, whereas significantly different values related to addition of heparin stand in italics (n=12-20).

## 4 Discussion

*S. aureus* has evolved a broad variety of virulence factors to successfully penetrate the host, evade the host’s immune system, multiply and spread unnoticed throughout the whole organism, which altogether can lead to life-threatening abscesses, bacteremia, endocarditis, sepsis, and septic shock. Staphylocoagulase, a protein capable of activating prothrombin directly, in a non-proteolytic manner, is one of the virulence factors, which are absolutely necessary for staphylococci to execute their pathogenic life cycle (1, 6). The SCG/ProT complex clots fibrinogen to fibrin like thrombin, but fails to reproduce many actions of thrombin, including the interaction with antithrombin, which leads to a virtually uncontrolled coagulase activity. This coagulase is not inhibited by heparins, vitamin K antagonists, factor Xa inhibitors. Since SCG occupies exosite I of thrombin, neither the bivalent hirudin derivatives can inhibit this coagulase (1). Thus, within the current spectrum of anticoagulants the direct thrombin inhibitors, such as dabigatran are the only current therapeutic tool against this coagulase-type virulence factors (1, 27, 28). The formation of a fibrin barrier around staphylococci provides a safe shelter to hide from immune cells, where the bacteria proliferate and at a later stage they express staphylokinase that activates the host fibrinolytic system to break down the fibrin wall and the staphylococcal colony spreads out (1). The host combats the bacteria using the innate immune system, which includes the activation of neutrophils, and release of NETs consisting of DNA, histones and a variety of further antimicrobial components (7, 29). *S. aureus* can also contribute to NET-formation by its virulence factors or kill neutrophils before they could release NETs (reviewed in Ref 30). Staphylococci are armed with virulence factors to survive the attacks of neutrophils, such as staphyloxanthin to capture reactive oxygen species and a nuclease to escape the released DNA-trap (1, 6, 12, 30, 31). Going under the cover of fibrin/fibronectin they have been shown to get endocytosed via integrin receptors in both endothelial cells and neutrophils and to survive intracellularly. In this way staphylococci can use neutrophils as transport vehicles to travel around the body and find vulnerable sites, such as damaged heart valves or seed metastatic abscesses (12, 32). In a murine model, where bacteremia and histamine-induced heart valve inflammation were used to provoke infective endocarditis, NETs were detected in infected cardiac vegetations, and absence of staphylocoagulase improved survival, decreased bacteremia, and increased NET-formation in the vegetations (29). The SCG-induced fibrin wall, which may also contain NET-components, has been shown to limit access of myeloid cells to bacteria inside the wall (33). Thus, studying the interplay of NETs and SCG-induced fibrin formation could improve our understanding of the consequences of currently applied anticoagulant therapy and emerging treatment modalities based on inhibition of NET formation (34) in the context of staphylococcal virulence.

We have recently described that SCG/ProT-induced fibrin and plasma clots are more prone to mechanical and fibrinolytic disintegration, which might contribute to the high rate of septic embolism from cardiac vegetations (13). The NET-components, histones and DNA have been shown to inhibit fibrinolysis, whereas H1 histones could mechanically strengthen the thrombin-induced fibrin structure (10, 13, 16, 35). H1 histones have been shown to incorporate into fibrin fibers and to favor the formation of thicker fibers. This fibrin network is characterized by an increased storage modulus (higher stress needed for the same deformation to occur), increased dynamic viscosity as shear stress increases, and significantly higher maximal bearable deformation before gel/fluid transition occurs and fibrin disintegrates. Our current data showed that incorporation of histones and DNA in the SCG/ProT-induced fibrin resulted in thicker fibrin fibers as in thrombin-induced fibrin (10), but also increased their structural heterogeneity reflected in the broader range of their size distribution (**Figure 1**; **Table 1**). These structural properties translated into a different mechanical impact. Neither DNA nor core histones or their combinations could improve the mechanical stability of the SCG/ProT-induced fibrin barrier. Only H1 histones, when applied at similar concentration under the same experimental conditions, as in our previous study (26), moderately strengthened the SCG/ProT-induced fibrin network, but at higher concentrations H1 also further destabilized the structure (**Figure 2**; **Table 2**). Thus, a conclusion can be drawn that if NETs are released at sites where SCG clots fibrinogen, a fibrin structure of weaker mechanical stability is formed with impaired barrier potency to help staphylococci in evading the host immune defense.

Both DNA and histones have been shown to inhibit fibrinolysis through non-covalent and covalent interactions with fibrin, serving as an alternative substrate of plasmin, and altering the native fibrin fiber geometry with consequent impairment of plasmin processivity in the course of fibrin degradation (14–16, 35). Our present data suggest that similarly to fibrin generated by thrombin, in the presence of histones, DNA, and combinations of DNA with histones the SCG/ProT-fibrin is less susceptible to lysis triggered by tPA than pure fibrin clots (**Figure 3**; **Table 3**). Although pre-formed plasma clots containing core histones alone could be lysed faster with tPA, incorporation of DNA or core histones combined with DNA rendered the clot more resistant to lysis than pure plasma clots (**Figure 4**; **Table 4**). Thus, concerning the *in vivo* situation when DNA-histone complexes are released during NET-formation, impaired fibrinolytic susceptibility of SCG-fibrin can be predicted. This implies that despite its mechanical fragility the SCG-coagulum containing NETs might be less prone to embolization than pure SCG-fibrin, if exposed to lytic agents.

Critically ill patients often receive anticoagulants to prevent thrombosis, and even if they had been taking a direct oral anticoagulant (thrombin, or factor Xa inhibitor) before hospitalization, low molecular-weight heparin is the preferred anticoagulant in intensive care units (36). In addition to their anticoagulant action, heparins are known to neutralize certain histone-mediated toxic effects and might reduce mortality among septic patients (37–39). On the other side of the histone-heparin interactions, histones have been shown to inhibit the anticoagulant effects of heparins (26, 40). Our present data indicate that SCG/ProT clots from plasma supplemented with unfractionated heparin, low-molecular-weight heparin or pentasaccharide are formed even faster than from native plasma, and NET-components cannot significantly change this tendency (**Table S3**). This observation is in line with previous findings that on the surface of catheter implants heparin promotes the formation of *S. aureus* biofilm composed of proteins, polysaccharides and extracellular DNA (41). In addition, one of the studied heparin variants (LMWH) accelerated the lysis of SCG/ProT-induced plasma clots containing histones or DNA alone, or their combinations (except for DNA+core histones) (**Table 4**). Two practical conclusions arise from these results: (i) faster SCG/ProT-induced clotting in patients with staphylococcal infection could help bacteria to evade the immune defense, whereas (ii) faster dissolution of SCG/ProT-induced fibrin in LMWH-treated patients with cardiac vegetations could promote septic embolism. Neither of these adverse effects of heparins is reversed by NETs. To improve survival in infective endocarditis, early diagnosis and identification of the specific bacteria enabling efficient antibiotic therapy and early surgical intervention have been suggested. Among the currently used anticoagulants the direct thrombin inhibitor, dabigatran, is the only one, which can inhibit staphylothrombin, and thereby limit the building of a protective fibrin barrier around the bacteria, moreover, its anticoagulant effect is not influenced by histones (1, 40). Staphylothrombin inhibition by dabigatran decreased *S. aureus* adherence to artificial surfaces and enhanced their killing by leukocytes *in vitro*, and reduced abscess size in a subcutaneous infection model (42). Inhibition of SCG-induced fibrin formation by neutralizing antibodies in murine septic models also improved survival rates (43). Targeting infective endocarditis in *in vivo* animal models, adjunctive dabigatran therapy has indeed reduced valve vegetation size, bacterial load in aortic valves, and pro-inflammatory markers, which supports the latest anti-biofilm approach to improve the outcome in *S. aureus* infections (44, 45).

As a concluding remark, our study demonstrated that if NETs are released when SCG generates fibrin, their major components (DNA-histone complexes) reduce the mechanical stability of the fibrin and probably compromise its function as a mechanical barrier that helps Staphylococci to evade the immune defense. In addition, the DNA-histone complexes render the SCG-fibrin more resistant to lysis and thereby less prone to embolization. However, these NET components cannot counteract the heparin effects to accelerate the formation of fibrin by SCG and its lysis.

## Data availability statement

The original contributions presented in the study are included in the article/**Supplementary material**. Further inquiries can be directed to the corresponding author.

## Author contributions

EK and VF performed the sample preparation for SEM, the kinetic, permeability, and rheometrical studies, analyzed the data and wrote the manuscript. LS performed the scanning electron microscopic work. SC contributed to the design and management of the permeability studies. CT designed and performed the expression of SCG, and along with KK designed the study, supervised the analysis and wrote the manuscript. All authors contributed to manuscript revision, read and approved the submitted version.
