# Supplementary material for: DNA and histones impair the mechanical stability and lytic susceptibility of fibrin formed by staphylocoagulase
Source: Front Immunol. 2023 Aug 17;14:1233128. doi: 10.3389/fimmu.2023.1233128 (PMC10470048; doi:10.3389/fimmu.2023.1233128)
Supplement: Supplementary file 1 [file DataSheet_1.pdf]

Supplementary Data

DNA and histones impair the mechanical stability and lytic susceptibility of fibrin formed by staphylocoagulase

Erzsébet Komorowicz[†], Veronika J. Farkas[†], László Szabó, Sophie Cherrington, Craig Thelwell, Krasimir Kolev*

†These authors contributed equally to this work and share first authorship

Correspondence:

*Krasimir Kolev, Semmelweis University, Department of Biochemistry and Molecular Biology, 1094 Budapest, Tűzoltó utca 37-47., Hungary, tel.: +36 1 4591500/60035, fax: +36 1 2670031, e-mail: Kolev.Krasimir@semmelweis.hu

Table S1. Structural characteristics of fibrin formed by thrombin in the presence of NET components. Fibrin clots containing histone H1 or core histone (75 µg/ml) alone or in combination with 50 µg/ml DNA were examined for fibrin fiber diameter (scanning electron microscopy, median (bottom-top quartile)), network porosity (fluid permeability, K_s Darcy constant 10^{-9} cm^2, mean[SEM]), and fiber mass/length ratio (maximal turbidity, A_{max}, mean(SD)). Asterisks and pound signs indicate $p<0.05$ according to Kolmogorov-Smirnov test in the comparison of fibrin containing NET-components to pure fibrin, and of histone-DNA to fibrin with histone alone, respectively, n=12-20.

	none	DNA	H1 histone	H1 histone + DNA	core histone	core histone + DNA
fiber diameter	59 (47-73)	67 (53-83)	69* (53-89)	66* (52-85)	59 (47-73)	68* (53-85)
K_s Darcy constant	3.52 (0.28)	6.29* (0.77)	9.45* (1.51)	3.64$^{\#}$ (0.40)	9.90* (0.42)	10.25* (2.04)
maximal turbidity	1 (0.02)	1.14* (0.01)	1.37* (0.03)	1.15*$^{\#}$ (0.05)	1.35* (0.06)	1.17*$^{\#}$ (0.02)

Table S2. NET-components prolong dissolution of both SCS/ProT-, and thrombin-mediated fibrin clots with incorporated tPA. Fibrin clots containing 15 nM plasminogen and the NET-components histone H1 or core histone (75 µg/ml) alone or in combination with 50 µg/ml DNA were clotted by the indicated enzyme in the presence of 1.3 nM tPA to induce intrinsic fibrinolysis. Lysis-time (LT50) was defined as the time needed to reach half maximal turbidity at the descending end of the curve, and presented in relative units compared to pure fibrin, as mean (SEM). Asterisks and pound signs indicate *p*<0.05 according to Kolmogorov-Smirnov test in the comparison of fibrin containing NET-components to pure fibrin, and of histone-DNA to fibrin with histone alone, respectively, n=12-20.

	none	DNA	H1 histone	H1 histone + DNA	core histone	core histone + DNA
Thrombin	1.00 (0.01)	1.11 (0.03)	0.91 (0.06)	**1.41*# (0.09)**	**2.16* (0.09)**	**1.89* (0.10)**
SCG/ProT	1.00 (0.02)	1.13 (0.06)	1.16* (0.03)	**1.40* (0.10)**	**1.56* (0.05)**	**1.41*# (2.04)**

Table S3 Clotting-time (CT50) and maximal turbidity (Amax) of pre-formed plasma clots containing NET-components and heparins. Plasma clots containing 1 µM plasminogen and 250 µg/ml H1 or core histone, or 100 µg/ml DNA, alone or combined were pre-formed in microplate wells in the absence or presence of heparins (2.5 µg/ml UFH or LMWH, or 0.5 µg/ml HPS). Development of clot structure was followed by turbidimetry for 60 min at 340 nm wavelength, by then all curves had reached their plateau, and maximal turbidity (Amax) could be determined. Clotting-time (CT50) was defined as the time needed to reach half-maximal turbidity. Values (mean (SEM)) are presented in relative units compared to clots without any additive. Asterisks indicate $p<0.05$ according to Kolmogorov-Smirnov test in the comparison of clots containing NET-components+/-heparins to the pure plasma clot, whereas pound signs indicate a $p<0.05$ significant heparin-effect in comparison to the corresponding heparin-free plasma/NET composite clot (n=12-20). The same clots were subjected to fibrinolysis induced with surface-applied tPA, representative curves and lysis-times are presented in Figure 4 and Table 4.

	none	DNA	H1 histone	H1 histone + DNA	core histone	core histone + DNA
**no heparin**						
CT50	1.00(0.03)	0.91*(0.02)	0.96(0.03)	1.03(0.04)	0.88*(0.03)	1.01(0.05)
Amax	1.00(0.01)	1.00(0.03)	0.93(0.01)	0.98(0.01)	0.77*(0.01)	0.90*(0.01)
**UFH**						
CT50	0.72*(0.03)	0.63*#(0.03)	0.85*#(0.02)	1.03(0.05)	0.73*#(0.03)	0.82*#(0.09)
Amax	0.75*(0.02)	0.72*#(0.02)	0.82*#(0.01)	1.00(0.01)	0.76*(0.01)	0.86*#(0.01)
**LMWH**						
CT50	0.90(0.02)	0.72*#(0.06)	0.93(0.01)	0.95(0.05)	0.96(0.06)	0.86*#(0.03)
Amax	1.00(0.01)	0.93(0.01)	1.06*#(0.02)	0.91*#(0.03)	0.81*(0.06)	0.95(0.03)
**HPS**						
CT50	0.72*(0.04)	0.78*#(0.05)	0.86*(0.01)	0.83*#(0.05)	0.57*#(0.05)	0.92(0.05)
Amax	0.89*(0.01)	0.91(0.02)	0.77*#(0.01)	0.76*#(0.01)	0.67*#(0.03)	0.90*(0.01)
